# Exploring Functional Connectivity in Chronic Spinal Cord Injury Patients With Neuropathic Pain Versus Without Neuropathic Pain

**DOI:** 10.1089/neur.2023.0070

**Published:** 2024-01-05

**Authors:** Shreya Mandloi, Mashaal Syed, Isaiah Ailes, Omid Shoraka, Benjamin Leiby, Jingya Miao, Sara Thalheimer, Joshua Heller, Feroze B. Mohamed, Ashwini Sharan, James Harrop, Laura Krisa, Mahdi Alizadeh

**Affiliations:** ^1^ Department of Neurological Surgery, Vickie and Jack Farber Institute for Neuroscience, Department of Radiology, Thomas Jefferson University, Philadelphia, Pennsylvania, USA.; ^2^ Thomas Jefferson Integrated Magnetic Resonance Imaging Center, Department of Radiology, Thomas Jefferson University, Philadelphia, Pennsylvania, USA.

**Keywords:** fMRI, functional connectivity, neuropathic pain, spinal cord injury

## Abstract

The great majority of spinal cord injury (SCI) patients have debilitating chronic pain. Despite decades of research, these pain pathways of neuropathic pain (NP) are unknown. SCI patients have been shown to have abnormal brain pain pathways. We hypothesize that SCI NP patients' pain matrix is altered compared to SCI patients without NP. This study examines the functional connectivity (FC) in SCI patients with moderate-severe chronic NP compared to SCI patients with mild-no NP. These groups were compared to control subjects. The Neuropathic Pain Questionnaire and neurological evaluation based on the International Standard Neurological Classification of SCI were utilized to define the severity and level of injury. Of the 10 SCI patients, 7 (48.6 ± 17.02 years old, 6 male and 1 female) indicated that they had NP and 3 did not have NP (39.33 ± 8.08 years old, 2 male and 1 female). Ten uninjured neurologically intact participants were used as controls (24.8 ± 4.61 years old, 5 male and 5 female). FC metrics were obtained from the comparisons of resting-state functional magnetic resonance imaging among our various groups (controls, SCI with NP, and SCI without NP). For each comparison, a region-of-interest (ROI)-to-ROI connectivity analysis was pursued, encompassing a total of 175 ROIs based on a customized atlas derived from the AAL3 atlas. The analysis accounted for covariates such as age and sex. To correct for multiple comparisons, a strict Bonferroni correction was applied with a significance level of *p* < 0.05/NROIs. When comparing SCI patients with moderate-to-severe pain to those with mild-to-no pain, specific thalamic nuclei had altered connections. These nuclei included: medial pulvinar; lateral pulvinar; medial geniculate nucleus; lateral geniculate nucleus; and mediodorsal magnocellular nucleus. There was increased FC between the lateral geniculate nucleus and the anteroventral nucleus in NP post-SCI. Our analysis additionally highlights the relationships between the frontal lobe and temporal lobe with pain. This study successfully identifies thalamic neuroplastic changes that occur in patients with SCI who develop NP. It additionally underscores the pain matrix and involvement of the frontal and temporal lobes as well. Our findings complement that the development of NP post-SCI involves cognitive, emotional, and behavioral influences.

## Introduction

Spinal cord injury (SCI) is an insult to the spinal cord that causes severe debilitating chronic pain. It is characterized by the loss of motor and sensory functions below the level of injury attributable to a disconnection between the afferent and efferent tracts from the spine to the cerebral cortex.^1^ The National Center for Spinal Cord Injury Statistics Facts and Figures in 2020 reported that ∼294,000 persons suffer from SCI with ∼17,810 new cases each year.^2^ SCI was recognized as incurable in 1700 BC and is still considered at the forefront of challenging medical conditions. Years of research have still left SCI patients without full neurological recovery.

A major complication in patients post-SCI is chronic pain. Chronic pain has been found in ∼18–63% of patients post-SCI.^3,4^ Chronic pain in SCI can be divided into two major classes: nociceptive pain and neuropathic pain (NP).^5^ Nociceptive pain is defined as pain that arises from actual or threatened damage to non-neural tissue and activation of nociceptors/free nerve endings.^6^ With nociceptive pain, activity is generated by normal pain mechanisms in the periphery nociceptors with cell bodies in the dorsal root ganglia. Nociceptive pain is secondary to the musculoskeletal effects of SCI that include overuse of the upper body, muscle atrophy/weakness, poor posture, and spasticity.^5^ NP, on the other hand, is defined as pain caused by a primary lesion or dysfunction of the nervous system.^6^ Unlike nociceptive pain, NP in SCI is determined as purely pathological and is a consequence of central nervous system (CNS) damage and inflammation instead of a protective response. NP in SCI patients is permanent and resistant to treatment and is further divided into at the level and below the level pain.^5^ Both at the level and below the level NP are described as hot-burning, tingling, pins and needles, shooting, squeezing, and electric shock.5 NP post-SCI can be spontaneous, but may also occur by extrinsic stimuli that are not normally painful or in an exaggerated form of noxious stimuli.^5^

Despite decades of research, the pain pathway of NP is unknown. Several lines of evidence show abnormal functioning of the brain in patients with SCI. SCI may induce changes in the balance of excitatory and inhibitory influences at the pain pathway.5 Additionally, there is decreased gray matter found in the anterior cingulate cortex, left insula, left secondary somatosensory cortex, and bilateral thalamus post-SCI.1,7 Alterations in gray matter can result from varying numbers of neurons, interneurons, glial cells, or a decreased size in cells overall. The decreased gray matter volume in pain-regulating areas like the somatosensory cortex and insula can be attributable to maladaptive plasticity and lead to chronic NP.^7^ Additionally, it has been found that allodynia in subjects with NP is correlated with signal changes in the primary and secondary somatosensory cortices, the insula and thalamus.^8^

Currently, there is no treatment that has been predictable, effective, and safe for long-term use in managing NP post-SCI. The standard protocol is the anticonvulsant drugs gabapentin and pregabalin or the antidepressant amitriptyline for these patients.^8^ However, pharmacological treatments for NP post-SCI are only partly successful at relieving pain and are often discontinued because of side effects that include: dry mouth, spasticity, trembling, dementia, blurred vision, weakness, and sleep disturbances.^8^

Functional magnetic resonance imaging (fMRI) is a non-invasive method of measuring neuronal activity in the human brain. Signals of fMRI arise from changes in neuronal activity, and it is generally assumed that the fMRI signal is proportional to a measure of local activity.^9^ fMRI measures neural activity by an indirect evaluation of changes in the hemodynamics of the capillary beds. fMRI can image the whole brain at the same time and uses algorithms to segregate functional circuits to understand the underlying pathophysiology of pain.^10–12^ Functional imaging has redefined chronic pain as a degenerative disease and has helped the scientific community better understand diseases such as fibromyalgia. fMRI has been used extensively to understand the transition of acute to chronic pain. Most of the work related to fMRI pain uses thermal stimuli to activate pain circuits. fMRI has helped identify brain regions involved in pain processing, with some of the regions being part of the well-defined pain circuit and others' role not fully understood.^10–12^ fMRI studies have also reported somatotopic organization of pain processing beyond the primary somatosensory cortex in areas such as the insula and trigeminal system. fMRI studies so far show that neuroimaging may be able to define a correlation between CNS activation and subjective pain scale questionnaires.^10–12^ Additionally, they show that neural systems in the pain response are complex and we still need a further understanding to manage many chronic pain conditions.

The lack of understanding of the pain pathway for NP post-SCI, as well as the lack of treatment options, underscores the need to gain deeper insight into the pain matrix. Understanding the pain matrix can help develop potential therapeutic targets. This study focuses on exploring FC in chronic SCI patients with NP versus without NP to better elucidate the plasticity of the brain. We hypothesize that patients with SCI and NP have different FC patterns compared to those with SCI without NP and healthy controls.

## Methods

### Participants

In this study, SCI patients with moderate-severe chronic NP were compared to SCI patients with mild-nNP and control subjects. For all healthy controls and SCI patients, age and sex were collected. The International Standards for Neurological Classification of Spinal Cord Injury was used to evaluate patients with SCI. Neurological level of injury is defined as the most caudal level of the spinal cord with intact sensation and movement against gravity. Additionally, a complete injury is defined as no sensation within the sacral spinal cord. From SCI patients specifically, American Spinal Injury Association scores, level of injury, and age of injury were collected. Medication use for SCI patients was collected as well. In order to classify patients as having NP, the Neuropathic Pain Questionnaire (NPQ) was used. The NPQ has been used for the initial screening of NP patients and has been proven to have high specificity and sensitivity in many chronic-pain–related studies.^13–15^ In order to differentiate whether patients had none-to-mild or moderate-to-severe NP, the pain rating scale was used. The pain rating scale is from 0 to 10, and 3 was used as the cut-off point. Those with none-to-mild NP had a pain scale number ≤3 whereas those with moderate-to-severe NP had a score >3. Pain management medications for those with SCI include acetaminophen, gabapentin, oxycodone, and medical marijuana. All methods were performed in accordance with the relevant guidelines and regulations approved by the institutional review board.

### Inclusion criteria

Only patients with NP or healthy controls were included in this study. None of the SCI patients included had symptomatic traumatic brain injury. Patients included were on a stable pain medication management with no changes of their pain medications during the study.

### Exclusion criteria

Patients with a baclofen pump and spinal cord stimulation implantation were excluded from the study because they were not compatible with scanning guidelines. Additionally, any other types of pain (nociceptive pain, nociplastic pain, etc.), as indicated by the NPQ, were excluded from the study.

### Image acquisition

All patients underwent resting-state fMRI (rsfMRI) scans before surgery using a 3.0T Ingenia Phillips scanner with a 32-channel head coil (Koninklijke Philips N.V., Amsterdam, The Netherlands). rsfMRI images were acquired axially using a single-shot echo planar imaging sequence in the same anatomical location prescribed for T1-weighted images. The T1-weighted imaging parameters used were: field of view (FOV) = 24.0 cm, voxel size = 1.0 × 1.0 × 1.0 mm^3^, matrix size = 512 × 512, repetition time (TR) = 12 ms, echo time (TE) = 6 ms, and slice thickness = 1 mm. Resting-state imaging parameters were FOV = 23.0 cm, voxel size = 3.5 × 3.5 × 3.5 mm^3^, matrix size = 128 × 128, TR = 2 sec, TE = 25 ms, number of averages = 1, and acquisition time = 12 min (360 volumes). Participants were instructed to relax, keep their eyes open, and think of nothing in particular during the resting-state scan.

### Data pre-processing

Pre-processing was carried out on the publicly available CONN FC toolbox in conjunction with SPM12, following the recommended processing pipeline. Structural and functional raw data were visually inspected for quality control. Next, functional images were corrected for temporal differences by slice timing; and for head motion by realignment and co-registration to the patient's T1-weighted scan. Co-registration and resampling of functional data to align with a reference image aim to mitigate distortion artifacts resulting from head movement. To further enhance data quality, two criteria were applied: 1) identifying excessive subject motion and 2) monitoring global blood-oxygen-level–dependent (BOLD) signal changes. Functional volumes with framewise displacement exceeding 0.9 mm or global BOLD signal variation surpassing 5 standard deviations were flagged as outliers and subsequently excluded from the data set. Data then were normalized to Montreal Neurological Institute space, spatially smoothed with an 8-mm full-width half-maximum Gaussian kernel, and denoised based on anatomical component analysis correction (aComCor) to avoid spurious results caused by increased sensitivity of rsfMRI to physiological and extraneous noise. In addition, resting-state time series were linearly detrended and bandpass filtered (0.008–0.090 Hz) to select for low-frequency components and reduce low-frequency drift, noise effects, and the confounding influence of respiratory (∼0.3 Hz) and cardiac (∼1 Hz) noise.

### Functional connectivity construction and statistical analysis

For each patient, whole-brain FC was constructed between a total of 175 brain regions of interest (ROIs) based upon a customized derivation of the Automated Anatomical Labeling Atlas 3 (AAL3). Our alterations to the traditional AAL3 atlas included additional regions such as the left and right pendunculopontine nucleus, left and right pontomedullary reticular formation, and brainstem. After the data pre-processing stage, the rsfMRI data underwent further analysis. Specifically, the BOLD signal at each time point (volume) from each pre-defined ROI was meticulously extracted. These data were then utilized to establish the FC between ROIs by computing Pearson's correlation coefficient between the time series extracted from each ROI. This process yielded a collection of correlation matrices for each subject, encapsulating the inter-regional connectivity patterns within the brain.

To assess significant differences in connectivity among distinct groups, namely 1) persons with SCI presenting moderate-to-severe chronic NP, 2) SCI persons without or with mild NP, and 3) healthy control subjects, a statistical approach was used. Two-sample *t*-tests were performed while controlling for potential confounding factors such as age at the time of enrollment and sex. Additionally, Benferroni's correction was applied to account for the multiple hypothesis testing.

## Results

### Distributions in demographics and clinical characteristics

A total of 10 participants with SCI were included. Based on the NPQ, 7 participants indicated that they had NP (48.6 ± 17.02 years old; 6 male and 1 female) whereas 3 participants did not (39.33 ± 8.08 years old; 2 male and 1 female). A total of 10 neurologically intact controls were included in this study (24.8 ± 4.61 years old; 5 male and 5 female). The Abbreviated Injury Scale (AIS) score is a standardized neurological examination score used to assess sensory and motor levels affected by SCI. All the participants of the study who had NP post-SCI were grades A and B. Two of the participants of the study who did not have NP post-SCI were grades D and C whereas the third participant who did not have NP post-SCI was grade A. Table 1 details the subject demographics of those with SCI including the neurological level of injury and severity of SCI.

**Table 1.** Demographics and Clinical Evaluations for Participants With SCI

Participant	Sex	Age (years)	Age at injury (years)	Cause of injury	Neurological level of injury	Severity of injury: AIS Grade	Presence (1) or absence (0) of neuropathic pain
1	F	27	18	MVA (restrained)	C6	B	1
2	M	53	51	MVA (unrestrained)	C3–C6	A	1
3	M	33	30	Fall	C5–C7	A	1
4	M	40	37	MVA (restrained)	C6	B	1
5	M	38	21	Fall	C7	C	0
6	M	77	39	MVA	T4	A	1
7	M	48	10	MVA	T5	A	0
8	F	32	8	Sports	C7	D	0
9	M	55	27	Fall	T12	A	1
10	M	54	48	MVA	T4	A	1

SCI, spinal cord injury; MVA, motor vehicle accident; AIS, AIS, Abbreviated Injury Scale.

### Global functional connectivity alterations in those with pain versus without pain

**FIG. 1.**
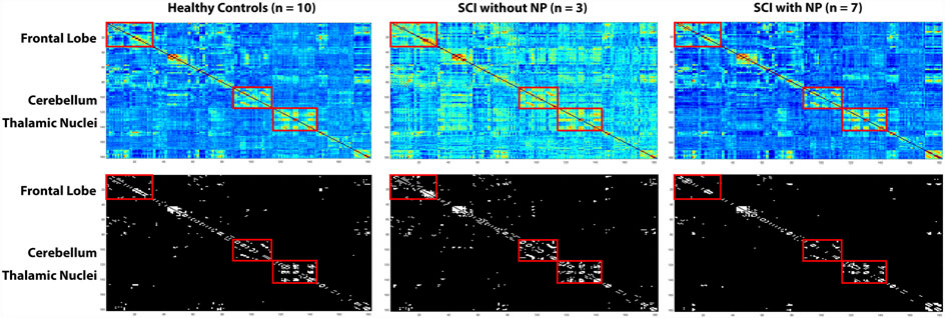
Averaged FC matrices of SCI patients with pain and without pain. There are visually pronounced connectivity differences in the frontal lobe, cerebellum, and thalamic nuclei indicating that patients with NP have globally distinct FC patterns compared to persons without NP. FC, functional connectivity; NP, neuropathic pain; SCI, spinal cord injury.

**FIG. 2.**
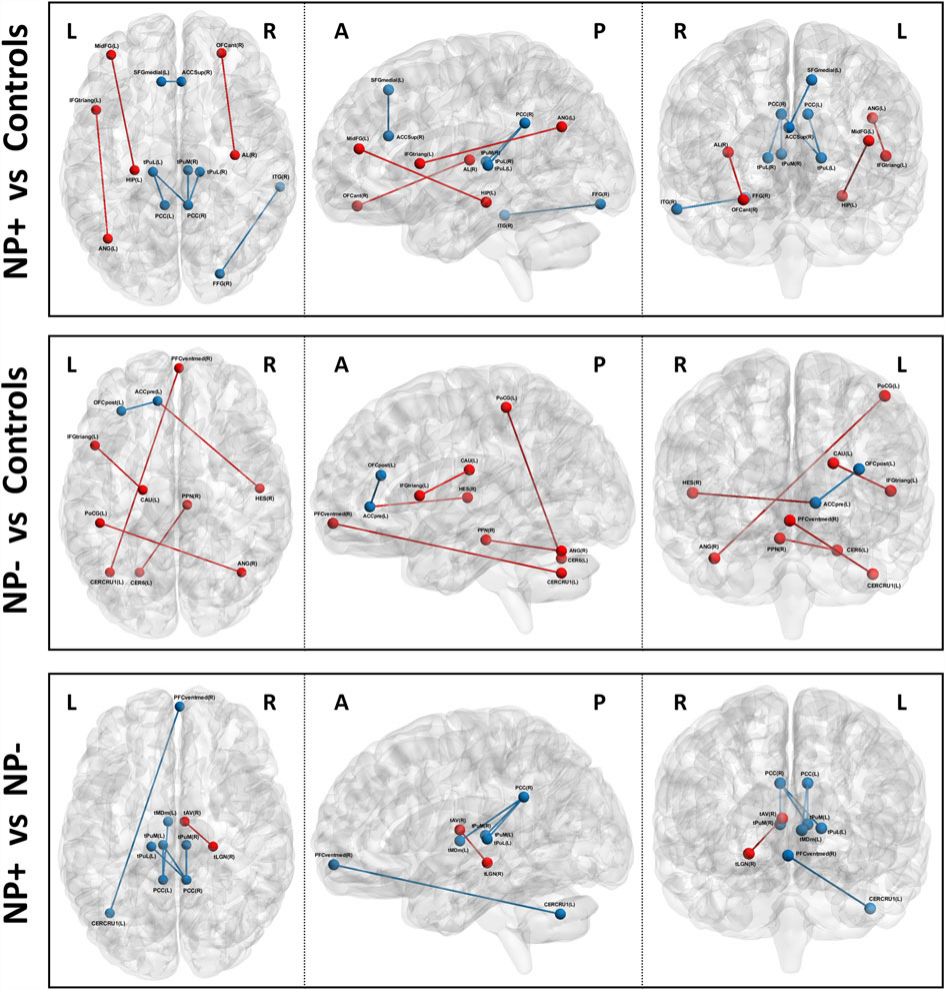
This figure is a functional connectivity map and represents NP patients compared to controls at the top panel, nNP patients to controls in the middle, and NP patients to nNP patients at the bottom panel. Red demonstrates increased connectivity, and blue demonstrates decreased connectivity. When comparing SCI participants with moderate-to-severe pain with those with no or mild and uninjured controls, specific regions were highlighted, mainly connections of thalamic nuclei (PuM, PuL, MGN, MDm, and LGN). When comparing SCI without pain with uninjured controls, increased connectivity of basal nuclei, brainstem nuclei, as well as sensory and motor cortices have been detected. When comparing NP patients to nNP patients, decreased connectivity is observed. NP, neuropathic pain; nNP, no NP; SCI, spinal cord injury.

Based on whole network analysis, there were distinct FC differences in those with NP post-SCI. Figure 1 represents the averaged FC matrices for the SCI patients with pain versus without pain showing that there are global FC differences between these two population groups. There were several distinctive connectivity patterns between the two groups in specific cortical and subcortical regions. The regions that highlight the most FC differences were the thalamic nuclei, frontal lobe, and cerebellum as demonstrated by the alteration in colors on the top of Figure 1 and the white on the black and white portion of Figure 1. Figure 2 represents an FC map of NP patients to controls, nNP patients to controls, and NP patients to nNP patients. When comparing SCI patients with moderate-to-severe pain to those with mild-to-no pain, specific thalamic nuclei had altered FC. These nuclei are: medial pulvinar (PuM); lateral pulvinar (PuL); medial geniculate nucleus (MGN); lateral geniculate nucleus (LGN); and mediodorsal magnocellular nucleus (MDm). When comparing SCI without pain to uninjured controls, hyperconnectivity could be observed in the basal nuclei, brainstem nuclei, and sensory and motor cortices.

### Thalamic involvement

A pairwise *t*-test was utilized to determine the FC alterations between persons with SCI and NP, those with SCI and nNP, and healthy controls, respectively. A positive *T*-value is indicative of greater FC between paired ROIs, whereas a negative *T*-value is suggestive of decreased FC.

**FIG. 3.**
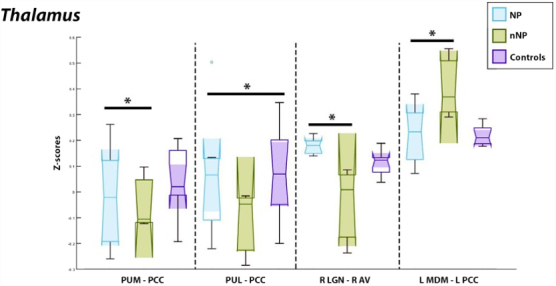
Significantly correlated regions of interest as related to the thalamus and its nuclei. Left, those with NP had experienced hyperactivity between the PuM and PCC compared to patients without neuropathic pain (nNP). Those with NP experienced hypoactivity between the R PuM/R PCC as well as between the L PUL/R PCC as compared to healthy controls visualized (left). Hypoactivation was also observed in the NP cohort between the R LGN/R AV and the L MDm/L PCC as compared to nNP patients visualized in the right two panels. L, left hemisphere; R, right hemisphere; PCC, posterior cingulate cortex; PuM, pulvinar medial; MDm, mediodorsal medial magnocellular; PuL, pulvinar lateral; LGN, lateral geniculate; AV, anteroventral nucleus.

Significant correlations were observed between the bilateral posterior cingulate cortex (PCC) and two thalamic ROIs; the bilateral medial PuM and bilateral lateral PuL. Those with NP exhibited hyperactivity between the PuM and PCC in comparison to those without NP (left PuM and left PCC: *p* = 7.20E-5, *T* = −5.30; left PuM and right PCC: *p* = 1.20E-4, *T* = −5.04; and right PuM and right PCC: *p* = 1.78E-4, *T* = −4.85). The significance can be visualized in Figure 3, which represents the averaged *z* scores. Those with NP were also found to experience hypoactivity as compared to healthy controls between the PuL and PCC (left PuL and left PCC: *p* = 1.32E-4, *T* = −5.00; left PuL and right PCC: *p* = 1.05E-4, *T* = −5.11; and right PuL and right PCC: *p* = 2.70E-4, *T* = −4.65). This can also be observed in Figure 3, which represents the averaged *z* scores between these thalamic nuclei and the subgroups.

Additionally, the left PCC was found to be significantly correlated with the left medial-dorsal medial magnocellular nucleus (MDm; *p* = 1.07E-4, *T* = −5.10). Between these ROIs, those with NP exhibited hypoactivity as compared to those without NP. Overall, the considerable connections from the PuM, PuL, and MDm to the PCC were especially noteworthy. Further, significance between the right lateral geniculate nucleus (LGN) and the right anteroventral nucleus (AV) was suggestive of hyperactivity in NP as compared to those without NP (*p* = 1.34E-4, *T* = 4.99). These can further be visualized on the right side of Figure 3.

### Frontal lobe involvement

**FIG. 4.**
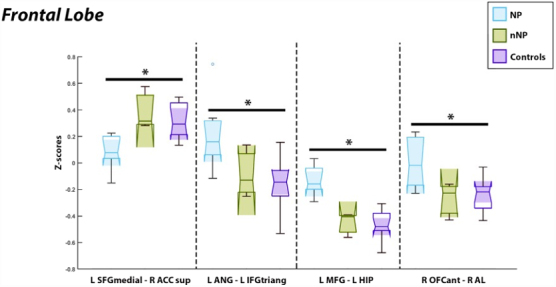
Significantly correlated regions of interest associated with the frontal lobe. Those with NP experienced hypoactivity between the L SFGmedial/R ACCsup as compared to healthy controls observed on the left. Hyperactivation was observed in the NP cohort between the L ANG/L IFG, L MFG/L HIP, and R OFCant/R AL as compared to healthy controls seen on the right three panels. L, left hemisphere; R, right hemisphere; IFGtriang, inferior frontal gyrus, triangular part; ANG, angular gyrus; SFGmedial, superior frontal gyrus, medial; ACCsup, anterior cingulate cortex, supracallosal; HIP, hippocampus; OFCant, anterior orbital gyrus; AL, insula anterior long gyrus.

Four paired ROIs with relationships to the frontal lobe were found to be significant. Hypoconnectivity was observed in patients with NP between the left medial superior frontal gyrus (SFGmedial) and right supracallosal anterior cingulate cortex (ACCsup) compared to healthy controls (*p* = 3.8E-5, *T* = −5.62). Patients with NP experienced hyperconnectivity between the left angular gyrus (ANG) and left inferior frontal gyrus (IFGtriang; *p* = 4.7E-5, *T* = 5.52), the right anterior orbital gyrus (OFCant) and right insula anterior long gyrus (AL; *p* = 7.8E-5, *T* = 5.26), and the left middle frontal gyrus (MFG) with the left hippocampus (HIP; *p* = 1.85E-4, *T* = 4.83) compared to healthy controls. Figure 4 demonstrates the hypoconnectivity observed between the SFGmedial and ACCsup in NP patients compared to healthy controls on the left and the hyperconnectivity between the LANG-LIFGtriang, LMFG-LHIP, and the R OFCant/RAL in NP patients compared to healthy controls on the right.

### Associations with the temporal lobe

**FIG. 5.**
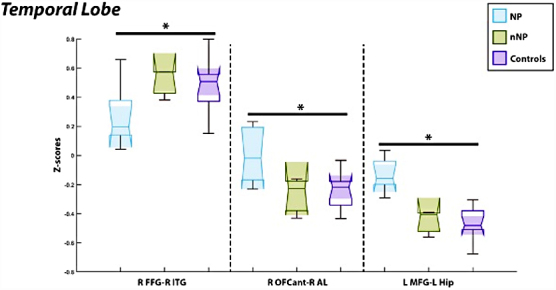
Comparisons of the functional connectivity between regions of interest as associated with the temporal lobe revealed hypoactivation in those with neuropathic pain as compared to healthy control subjects between the R FFG/R ITG seen on the left. Conversely, those with neuropathic pain experienced hyperactivation between both the R OFCant/R AL and the L MFG/L HIP in comparison to healthy controls seen on the right. L, left hemisphere; R, right hemisphere; FFG, fusiform gyrus; ITG, inferior temporal gyrus; OFCant, anterior orbital gyrus; AL, insula anterior long gyrus; MFG, middle frontal gyrus; HIP, hippocampus.

As described above, in relation to the frontal lobe, relevant connections in the temporal lobe also included the associations between the right anterior orbital gyrus (OFCant) and right insula anterior long gyrus (AL) and the left middle frontal gyrus (MFG) with the left hippocampus (HIP) Patients with NP experienced hyperconnectivity between the right anterior OFCant and RAL (*p* = 7.8E-5, *T* = 5.26) compared to healthy controls. Additionally, patients with NP experienced hyperconnectivity between the LMFG and LHIP (*p* = 1.85E-4, *T* = 4.83) compared to healthy controls visualized on the left of Figure 5. Additionally, patients with neuropathic pain had hypoconnectivity between the right fusiform gyrus and right inferior temporal gyrus (*p* = 2.64E-4, *T* = −4.65) compared to healthy controls, visualized on the left of Figure 5.

## Discussion

SCI causes a disruption in the neural circuitry throughout both the brain and body, leading to a permanent neurological disability.^16^ Around 61% of patients with SCI report chronic NP as a complication,^17^ yet the plasticity changes in patients with NP post-SCI and the extent of reorganization remain unknown. Having a deeper understanding into the changes that occur in this patient population could potentially contribute to the development of focused treatment options. This study suggests that there are alterations to the FC patterns and therefore changes in the neural plasticity in patients with NP post-SCI as differentiated by their non-NP counterparts. Notable FC alterations were determined to be related to thalamic nuclei, the frontal lobe, and the temporal lobe.

### Alterations of thalamic connectivity

The thalamus has been described as a key subcortical structure in the transmission of nociceptive input to the cerebral cortex.^18^ As such, it is believed to play a considerably centralized role in the development of NP.^19,20^ Structurally, a decrease in thalamic gray matter volume has been associated with chronic NP, whereas a reduction of gamma-aminobutyric acid content may contribute to altered thalamic connectivity patterns.^19^ Disturbances in thalamocortical activity may, in particular, be implicated in the constant perception of pain, as evidenced by studies characterizing its involvement in a variety of chronic pain conditions not limited to fibromyalgia, post-herpetic neuralgia, trigeminal neuropathy, and chronic low back pain.^19–23^

In fact, through electrophysiological data, Anderson and colleagues observed neuroplastic changes in the functional properties of thalamic neurons in patients with SCI-induced chronic pain.^24^ When comparing SCI-inflicted persons with moderate-to-severe NP to those with mild-to-nNP and healthy controls, this work also demonstrated differences in FC associated with the PCC and a particular subset of thalamic nuclei, including both the medial and lateral pulvinar nuclei, and the mediodorsal medial magnocellular nucleus. Although the former pair is most commonly associated with saccadic eye movement and visual attention, the latter in particular has been involved in distinguishing ascending signals evoked by noxious mechanical stimulation.^18^ You and colleagues further described this nucleus as capable of triggering a descending facilitatory pathway that induces secondary mechanical hyperalgesia, without accompanying heat hypersensitivity.^18^

Although the reduced FC in thalamic nuclei has been linked to an abnormal dysregulation of the inhibition of pain,^21,22^ the PCC has been previously implicated in the overall perception of pain, affecting several aspects of pain processing and regulation.^18,20^ Although our results demonstrated a general decrease in thalamus-related FC in those with NP post-SCI in comparison to healthy controls, similar to what has been described in cases of fibromyalgia, Li and colleagues found an increase in FC patterns between the thalamus and PCC in persons with chronic low back pain.^20^ The variation between our outcomes may be suggestive of disease-state–specific FC alterations between the thalamus and nearby neural structures; regardless, our work coincides with the overall hypothesis that chronic NP is associated with altered thalamic anatomy and activity, lending itself to generalized sensory abnormalities commonly experienced with the condition.^25,26^

Additionally, our results depicted increased activation between the lateral geniculate nucleus and anteroventral nucleus in those with NP post-SCI, as compared to those without NP. Thus far, investigations related to the functional connections of the lateral geniculate nucleus, a thalamic nuclei primarily responsible for visual processing, has been limited to the pathogenesis of migraine as related to photophobia.^27^ The underlying aberrant functional alterations between the lateral geniculate nucleus and structures in both the pain perception regulatory network and the emotion regulation network may augment its clinical manifestation.^27^ The anteroventral nucleus, on the other hand, has been determined to contribute to spatial learning, memory, cognitive processes, and attention.^28^ In part, these facets may be attributed to this nuclei's projections to the limbic cortex, a region not only implicated in memory and spatial learning, but is also related to the perception of affective aspects of pain similar to the PCC.^18,29^

In their description of neural networks that comprise the pain connectome, Kucyi and colleagues characterized pain as being a multi-faceted ailment inter-related with cognition and attention.^30^ Pain and attention mutually influence one other; although the salience of pain is in itself attention-demanding, engaging in other attention-demanding tasks, stimuli, and thoughts can alter the quality of pain and overall neural processing of nociceptive input.^30^ In considering that significant thalamic nuclei recognized in our study such as the anteroventral nucleus, as well as the medial and lateral pulvinar nucleus, are also known to hold roles related to attention, our findings by and large are supported by this concept.

### Alterations of frontal lobe and temporal lobe-related functional connectivity patterns

The inter-relations of pain and attention are further emphasized in our findings affiliated with regions in both the frontal lobe and temporal lobe. Aside from its well-known feature of cognition and working memory, Seifert and Maihofner explained the frontal lobe's capabilities of modulating one's ability to feel pain and its role in central sensitization.^31^ Moreover, evidence from both basic and clinical science has shown that cognitive dysfunction can be induced by NP.^32^

Our analysis revealed increased FC between the left angular gyrus and left inferior frontal gyrus pars triangularis in those with NP as compared to healthy controls. Similar to a few of the thalamic nuclei mentioned previously, the angular gyrus has also been documented to play a role in attention, spatial memory, and cognition.^33^ The pars triangularis, however, has been thus far considered from the perspective of language processing. Further elucidation of the impacts of hyperactivity existing between these two ROIs would likely be best explored through neuropsychological testing of our NP patients; for example, the Logical Memory subtests I and II of the Wechsler Memory Scale (WMS)-III, specifically for the investigation of verbal memory decline, and the spatial span subtest of the WMS-III to measure short-term spatial memory capacity.^34^ Interestingly, in their investigation of 38 age-, sex-, and education-matched chronic pain patients, Moriarty and colleagues determined that those with chronic pain had a lower estimated IQ than healthy controls, and specifically showed impairments on measures of spatial and verbal memory.^34^ Granted, their participant demographics did not explicitly recount these persons as having endured SCI, and perhaps disease-state specifics may account for the discrepancies between our findings.

Our analysis also conveyed that those with NP experienced hypoactivity as compared to healthy controls between the left middle superior frontal gyrus and right supracallosal anterior cingulate cortex. The anterior cingulate cortex is implicated in the perception of affective aspects of pain, as well as pain vigilance, attention, and awareness; most frequently, biochemical deficits associated with this structure have been likened to chronic-pain–related anxiety, depression, behavioral sensitization, and hyperalgesia.^35–38^ In combination with the superior frontal gyrus, a region associated with a variety of cognitive and memory-based tasks, the observed decrease in FC between these two regions in those with chronic NP may coincide with the concommittant emotional and mood disorders frequently experienced by this patient population.^18,39–41^ Comparable to the mutually influential relationship between pain and attention is the relationship between pain and anticipation. This affiliation may arise from deficits in the connectivity patterns of the anterior cingulate cortex. For example, it appears that even the expectation of pain can evoke the activation of cortical areas underlying pain-related affect in this population, resembling that of a real pain experience.^42,43^ Conversely, past pain experiences can strongly affect pain anticipation and lend itself to associated network activation patterns.

Our findings also revealed hyperactivation in those with NP as compared to healthy controls between the right anterior orbital gyrus and right insula anterior long gyrus, as well as the left middle frontal gyrus with the left hippocampus. Once again, the connections to regions in the orbital cortex, insular cortex, and limbic cortex have all been related to the perception of affective aspects of pain.^18^ In their summarized account of the structural and functional changes in pain chronification, McCarberg and Peppin further assert the relevance of spatiotemporal reorganization from sensory to emotional and limbic structures during the transition from acute to chronic pain, as in accordance to longitudinal imaging studies.^44^ For one, it appears that the limbic-cortical network determines whether nociceptive signals are transient or chronic, by either diminishing or amplifying pathways that carry the emotional component of nociceptive inputs. Accordingly, there seems to be a strong association, both anatomically and mechanistically, between memory and the development of chronic pain; from one perspective, chronic pain can be seen as the persistence of the memory of pain, or the inability to extinguish painful memories.^44^ Likewise, memory retrieval of unpleasant experiences may also be associated with prolonged chronic pain conditions.^38^

Similarly, the temporal lobe also houses unique regions involved in the processing of chronic pain. In our work, significant hyperactivity was determined between the right fusiform gyrus and right inferior temporal gyrus in those with NP post-SCI, as compared to healthy controls. The functions of the fusiform gyrus are commonly ascribed to face perception, object recognition, and reading; however, there is evidence of its contribution to various aspects of memory, multi-sensory integration, and perceptual expertise.^45^ The inferior temporal gyrus also plays a key role in the ventral visual pathway and, similar to the fusiform gyrus, has been implicated in object, face, and scene perception.^46^ The isolation of these ROIs with significant alterations to the FC between them may support, in part, the hypothesis of Shimo and colleagues, which expressed that visualization of a painful event may trigger painful memories, thus provoking the affective dimension of pain.^38^ Previously, Ogino and colleagues also reported that even the imagination of pain even without physical injury engages the cortical representations of the pain-related neural network.^47^

All in all, our findings from the interrogation of FC patterns in those with NP post-SCI support the understanding detailed across the literature that pain pathways represent a complex sensory system with cognitive, emotional, and behavioral influences.

### Limitations

To the best of our knowledge, this is the first attempt to focus on investigating what specific neural structures are altered in SCI with NP. Though this study yielded several novel results, there are limitations to the study that must be considered. Limitations associated with FC include that FC refers to relationships that could potentially exist between neuronal populations without referencing physical connections. A limitation inherent to FC studies is that the differences may not be explained by pain alone. Given that this is a novel study that explores the FC differences between patients with SCI and NP and those with SCI without NP, we emphasize that baseline changes between these groups do exist. Further work is needed on larger populations to address whether these FC differences are inherent to the pain mechanism itself. Another limitation of the study is the relatively small sample size of patients included. Many patients with SCI have issues with spasticity and so cannot lay still for imaging. Additionally, many have transportation issues, so participating in a study that requires follow-up is difficult. Future studies should encompass a larger sample size of all subpopulations explored in this study. These weaknesses do not take away from the observation that there are neuronal firing differences in those who develop chronic NP post-SCI.

## Conclusion

This study successfully identified neuroplastic changes that occur in patients with SCI who develop NP. The findings imply that there are specific thalamic nuclei and areas of the cerebral cortex that have altered connectivity in those with NP and SCI compared to those with SCI without NP. Developing a further understanding subsequent to this preliminary study is key to develop targeted therapeutic targets for those with SCI and NP.

## Abbreviations Used

AAL3: Automated Anatomical Labeling Atlas 3
AIS: Abbreviated Injury Scale
BOLD: blood-oxygen-level–dependent
CNS: central nervous system
FC: functional connectivity
fMRI: functional MRI
FOV: field of view
MRI: magnetic resonance imaging
nNP: no neuropathic pain
NP: neuropathic pain
NPQ: Neuropathic Pain Questionnaire
PCC: posterior cingulate cortex
ROI: region of interest
rsfMRI: resting-state fMRI
SCI: spinal cord injury
TE: echo time
TR: repetition time
WMS: Wechsler Memory Scale
